# Antimicrobial Probiotics Reduce *Salmonella enterica* in Turkey Gastrointestinal Tracts

**DOI:** 10.1038/srep40695

**Published:** 2017-01-17

**Authors:** Brittany Forkus, Seth Ritter, Michail Vlysidis, Kathryn Geldart, Yiannis N. Kaznessis

**Affiliations:** 1 Department of Chemical Engineering and Materials Science University of Minnesota, Minneapolis, MN 55455, USA

## Abstract

Despite the arsenal of technologies employed to control foodborne nontyphoidal *Salmonella* (NTS), infections have not declined in decades. Poultry is the primary source of NTS outbreaks, as well as the fastest growing meat sector worldwide. With recent FDA rules for phasing-out antibiotics in animal production, pressure is mounting to develop new pathogen reduction strategies. We report on a technology to reduce *Salmonella enteritidis* in poultry. We engineered probiotic *E. coli* Nissle 1917, to express and secrete the antimicrobial peptide, Microcin J25. Using *in vitro* experiments and an animal model of 300 turkeys, we establish the efficacy of this technology. *Salmonella* more rapidly clear the ceca of birds administered the modified probiotic than other treatment groups. Approximately 97% lower *Salmonella* carriage is measured in a treated group, 14 days post-*Salmonella* challenge. Probiotic bacteria are generally regarded as safe to consume, are bile-resistant and can plausibly be modified to produce a panoply of antimicrobial peptides now known. The reported systems may provide a foundation for platforms to launch antimicrobials against gastrointestinal tract pathogens, including ones that are multi-drug resistant.

Foodborne gastrointestinal (GI) tract infections exact a vast global health toll, with nearly one in ten individuals falling ill each year^1^,^2^,^3^,^4^,^5^. In the U.S., non-typhoidal *Salmonella* (NTS) is responsible for the highest incidence of foodborne disease among bacterial pathogens, causing one million infections, 19,000 hospitalizations and over 400 deaths annually^2^,^6^.

Poultry is a major reservoir for NTS, with more than half of outbreaks linked to the consumption of contaminated poultry products^2^. In particular, *Salmonella enterica* serovar Enteritidis (SE) is the most common NTS strain in the U.S. food supply^2^. Poultry are asymptomatic carriers of SE, which allows rapid transmission through flocks. Subsequent spread to the community can occur at many stages along the food-production chain, but primarily at the consumption level^7^.

A related public health concern is the continuing emergence of antibiotic-resistant foodborne pathogens. The Centers for Disease Control and Prevention have estimated that 5% of NTS infections are already resistant to 5 or more antibiotics, and have classified NTS as a ‘serious threat’ to public health^6^. Resistant infections complicate patient treatment leading to prolonged illnesses, increased mortality rates, and higher medical expenses^6^.

This widespread resistance development is partly attributed to the heavy use of antibiotics in animal production^8^. Over 70% of the antibiotics produced in the U.S. are incorporated in livestock feed^9^. It is plausible that this continuous, sub-therapeutic administration applies selective pressures that facilitate the evolution of resistance development. Resistant strains may then be released to the environment through fecal shedding, human handling, and consumed foods^8^. This microbial release is concerning because there is considerable overlap between the antibiotics listed as ‘critically important’ by the World Health Organizations for human and animal health.

With these concerns, pressure is mounting to phase out the nontherapeutic use of antibiotics in U.S. food production^4^. Proposals to legislate feed-grade antibiotic removal have been met with significant opposition because a complete ban could lead to increased food prices and strain current agricultural practices^8^. Instead, the FDA issued a rule on livestock use with the agreement of animal pharmaceutical companies. According to this plan, drug companies will voluntarily revise the FDA-approved labeled use conditions, and change the marketing status from over-the-counter to Veterinary Feed Directive for drugs administered through feed or to prescription status for drugs administered through water. The ultimate goal is to promote the judicious use of medically important antimicrobial drugs in food animals and to remove the use of antimicrobial drugs for production purposes^4^.

Antibiotics in animal feed prevent or reduce the incidence of infectious disease^8^. Therefore, it may be surmised that with the imminent phasing-out process, alternative, affordable pathogen reduction technologies are needed to help mitigate consumer risk and exposure to foodborne pathogens.

We present tests of a modified probiotic *Escherichia coli* strain, Nissle 1917 (EcN), with the capacity to reduce SE counts in the GI tract of turkeys. Using recombinant DNA technology, we modified EcN to produce and secrete the antimicrobial peptide (AMP), Microcin J25 (MccJ25). We show in two repeat studies that the modified probiotic (EcN(J25)) can substantially reduce SE counts in the ceca of turkeys. With the administration of a single dose, we observe markedly improved SE clearance rates over a two-week period compared to treatment with the antibiotic, enrofloxacin, or the unmodified EcN.

Beneficial bacteria have been used in the agricultural industry for years to improve animal health and limit pathogen colonization. Often administered as competitive exclusion products, commercial treatments have been developed that are routinely administered to newly hatched birds^10^. Probiotic formulations have also been tested as feed additives. When incorporated in livestock diets, probiotics can improve animal growth, feed conversion efficiency^11^, and reduce shedding of enteric pathogens^12^.

Herein we show in two repeat trials that a modified probiotic (EcN(J25)) can substantially reduce SE counts in the GI tract of turkey poults. We observe markedly improved SE clearance rates over a two-week period compared to the traditional antibiotic enrofloxacin or the unmodified EcN.

## Design of system for AMP production and secretion

Our objective is to lower SE in the GI tract of poultry by employing probiotic EcN as the production and delivery vehicle of MccJ25. MccJ25 is a 21-residue peptide^13^ natively secreted from the human *E. coli* isolate AY25^14^. MccJ25 elicits a strong antagonistic affect against SE. Mature MccJ25 forms a lasso structure^13^, affording remarkable stability against unfolding and degradation. The peptide inhibits bacterial growth primarily by binding to RNA polymerase and obstructing nucleotide uptake^15^.

**Figure 1:**
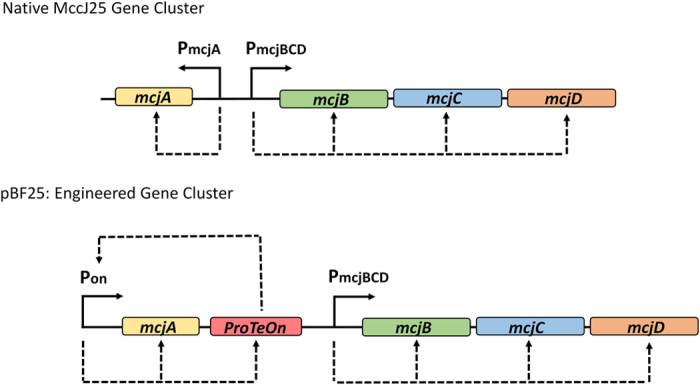
Schematic of the native and engineered MccJ25 operons. In the natural gene cluster, *mcjA* is divergently expressed from its dedicated processing and transport enzymes^16^. Expression of *mcjA* is activated in stationary phase^17^ while *mcjBCD* are constitutively expressed by a σ^70^-like promoter. pBF25 is the engineered construct used in this study where *mcjA* production is under the control of the ProTeOn+ system and all genes are convergently expressed.

The four genes facilitating production of the microcin, *mcjA, mcjB, mcjC*, and *mcjD*, are adjacently located on a native plasmid-borne operon (Fig. 1)^14^. mcjA encodes the MccJ25 precursor that is processed into the active form by enzymes, McjB and McjC^16^. The immunity protein, mcjD, enables the active efflux of the peptide to the extracellular space^16^. Natively, mcjA expression is governed by an ill-defined promoter that initiates production upon entry into stationary phase, with maximal expression in conditions of nutrient depletion. The other genes are hypothesized to be constitutively expressed^17^.

In this work, we engineered a strong promoter system to bypass native limitations on mcjA production. Expanding upon the synthetic ProTeOn system, a hybrid protein-promoter pair developed by Volzing and co-workers^18^, we designed ‘ProTeOn+’, a new genetic circuit, which enables constitutive, high-level production of downstream genes. ProTeOn is a synthetic activator protein constructed by physically linking the reverse tetracycline repressor protein to the activating domain of the LuxR transcription factor of *Vibrio fischeri*. ProTeOn makes strong contacts within the engineered DNA promoter site (Pon), which contains optimally spaced tetracycline and LuxR operator binding regions. This system recruits RNA polymerase and strengthens the holoenzyme-DNA interactions to strongly up-regulate gene expression^18^.

In ProTeOn+, we incorporated the ProTeOn protein in a positive-feedback control loop, allowing it to amplify its own production while driving expression of a target protein (Fig. 1). We incorporated ProTeOn+ upstream of mcjA and reorganized the genes to be convergently expressed, creating pBF25 (Supplemental Fig. 1). This system achieves more robust gene expression than previously afforded, and has a markedly stronger expression capacity than the strong, commercial promoter, OXB20 (Supplemental Figs 2 and 3).

## Results

### *In vitro* SE growth inhibition

**Figure 2:**
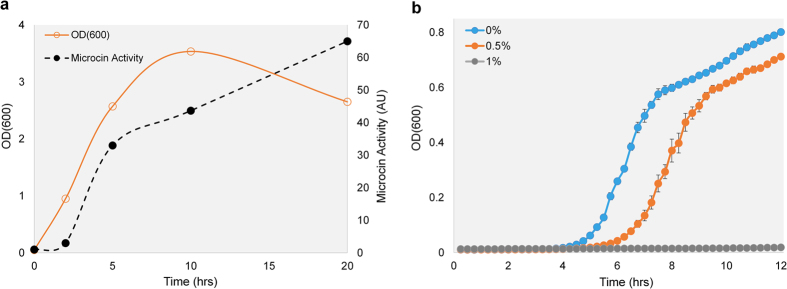
Modified probiotic elicits strong antagonistic activity against SE. (**a**) pBF25 enables growth phase independent production of MccJ25. The growth (OD600) of EcN(J25) is shown as a function of time. Supernatant was collected from EcN(J25) at 0, 2, 5, 10 and 20hrs following inoculation in fresh media. Two-fold serial dilutions of the supernatant were plated on SE-agar plates and the reciprocal of the final dilution with a visible halo was denoted as the activity units (AU). Microcin production from the modified Nissle commences immediately upon inoculation with visible activity after just 2 hrs. (The connecting lines used are for visualization purposes and are not interpolations of the data points.) (**b**) Kinetic growth inhibition of SE in the presence of the modified probiotic’s supernatant. SE is grown in the presence of 0, 0.5 and 1% volume fractions of supernatant collected from EcN(J25) demonstrating a strong antimicrobial effect.

To evaluate the antimicrobial potency of the engineered gene expression cassette, pBF25 was expressed in Nissle (EcN(J25)). As shown in Fig. 2a, EcN(J25) exhibits clear microcin activity after just 2 hours of culture growth and the peptide continues to accumulate in the supernatant over time. In contrast to the native promoter, the ProTeOn+ system affords microcin production across all stages of growth.

Figure 2b demonstrates that microcin-containing supernatant (SN) produced by EcN(J25) inhibits SE growth. The assay shows kinetic growth curves of SE in the presence of small volume fractions of MccJ25-rich SN. The two microcin containing treatments (0.5 and 1%) exhibit considerable growth suppression compared to the control (0%) after just 4 hours of exposure (p < 0.05).

### SE reduction in turkey ceca following EcN(J25) treatment

To test the efficacy of the modified probiotic at reducing colonization of SE in the GI tract, we performed two independent treatment trials in turkey poults. We focused on pathogen clearance in the ceca because they are the primary area of bacterial residence within the poultry GI tract, harboring over 10E10 organisms per gram of digesta^19^. The function of the ceca is to provide a stable bacterial ecosystem, and aid in feed fermentation and digestive processes. The ceca are the principal colonization site of *Salmonella* and are a major source of contamination in processing facilities^20^,^21^. Reducing *Salmonella* levels in the ceca at the pre-harvest stage may decrease the amount that initially enters the food chain.

We challenged turkey poults at 4 days post-hatch with a 1 ml oral gavage of 10E7 colony forming units (CFU) of SE (day 0), followed on day 1 by treatment with 1 ml of either 10E7 CFU EcN, 10E7 CFU EcN(J25), or PBS (phosphate buffer saline). These primary treatment groups allowed for comparison between the modified probiotic’s engineered antimicrobial activity and any inherent competitive exclusion or native antagonistic effect of the unmodified strain. The SE strain used, MH91989, was previously isolated from a chicken GI tract and is known to colonize poultry intestines.

**Figure 3:**
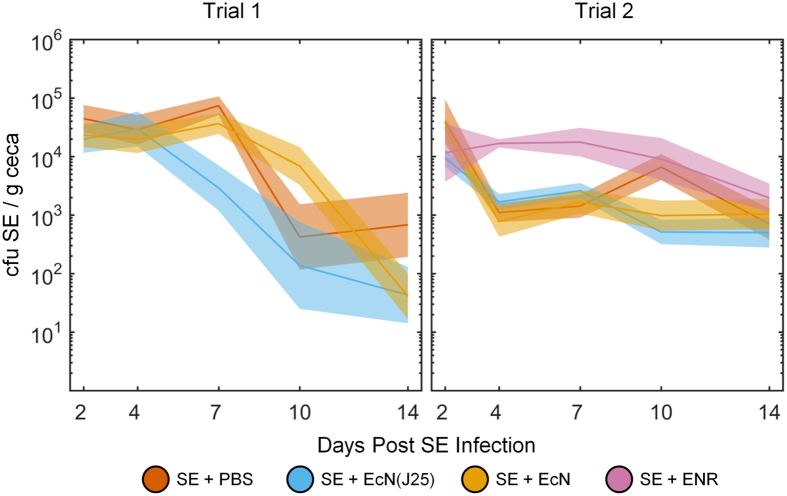
SE reduction in the ceca of turkey poults administered the EcN(J25) treatment. Turkey poults at 4 days post-hatch (day 0) were challenged with 10E7 CFU of SE. On day 1, birds were treated with PBS, EcN(J25), EcN, or ENR (Trial 2 only). 3–8 birds were euthanized from each treatment group 2, 4, 7, 10, and 14 days post-infection. SE and EcN were enumerated using selective plating to determine CFU per gram of ceca. The average SE counts at each necropsy point are shown for each treatment group at each time point for both trials. Time course line thickness represents the standard error. Both trials were analyzed separately using ANOVA and post-hoc analysis was performed for pairwise comparisons. More detailed descriptions of the statistical analysis can be found in the Materials and Methods.

Over a two-week period, we extracted the ceca from birds at 5 time points post SE-infection and enumerated the SE and Nissle count densities. Figure 3 shows the trajectories of the SE counts for each individual treatment group throughout each of the studies. In both trials, all SE counts decline for all groups over the two-week trial.

In Trial 1, the SE counts in the EcN(J25)-treated birds were significantly reduced compared to the untreated birds (p = 0.03, see Materials and Methods). By the final time point (day 14), the SE counts were reduced by over an order of magnitude, with SE appearing at 25x lower levels than the SE-control group. Trajectories displaying individual bird counts for both challenge strains can be found in Supplemental Fig. 4.

In Trial 2 no such significant reduction was observed (p = 0.27, see Materials and Methods). The differences between the SE-reduction trends in the two trials may be attributed to slightly different experimental parameters. In Trial 2, the crinoline flooring in the isolator units was removed on day 1 to mitigate any reinfection process caused by coprofagia. In Trial 1 this flooring was removed on day 4. The earlier removal in Trial 2 may explain the rapid 10x reduction of SE observed at the first collection point in the EcN(J25)-birds, absent in Trial 1. We note that fecal shedding within housing facilities and subsequent ingestion have been implicated as the primary route of horizontal pathogen transmission^22^. In Trial 2, all treatment groups, excluding the ENR-group, experience a rapid decrease in SE counts by day 4, likely because of pathogen shedding in a potentially cleaner environment.

In both trials, the unmodified Nissle did not by itself yield a significant reduction in SE counts. This result allows us to suggest that MccJ25 activity was responsible for the faster clearance rate from the ceca observed in Trial 1.

In Trial 2, we compared the efficacy of the EcN(J25) treatment to the activity of a single dose of enrofloxacin (ENR), a fluoroquinolone antibiotic. As shown in Fig. 3, the ENR treatment led to significantly higher SE levels than all other treatment groups (p ≤ 0.02, see Materials and Methods).

To monitor animal health and assess any adverse effects of the probiotic treatments, we included a PBS control group, as well as a group administered EcN(J25) in the absence of SE. These control treatments enabled us to examine cecal score and bird weight as indicators of overall health. All treatments showed no adverse effects, and bird weight remained nearly identical across all groups (Supplemental Fig. 5). In addition, we observed no visible changes between any of the groups in the morphology of the GI tract and the nature of the cecal contents.

The probiotic does not colonize the intestinal tract as strongly as SE. The levels of the modified and unmodified probiotic were monitored throughout the course of the study. EcN and EcN(J25) passed through the intestines with a residence time of 3–5 days before counts dropped below the level of detection (Supplemental Fig. 6).

It is interesting to note that no resistance development to the microcin was observed in any of the tested SE isolates following their passage to the ceca (Supplemental Fig. 7).

### Microbiome analysis

The poultry GI tract is developmentally very active in the early period following hatch. Intestinal health is critical to bird development and for years commercial poultry producers have used antibiotics to achieve and maintain GI stability and consistency within flocks^23^. Previous studies suggest that EcN enhances early GI tract maturation^24^, reduces shedding of enteric pathogens, and may improve body weight gain^25^. We performed a microbiome analysis on 258 ceca samples to ensure that our modification on Nissle did not adversely affect intestinal health.

**Figure 4:**
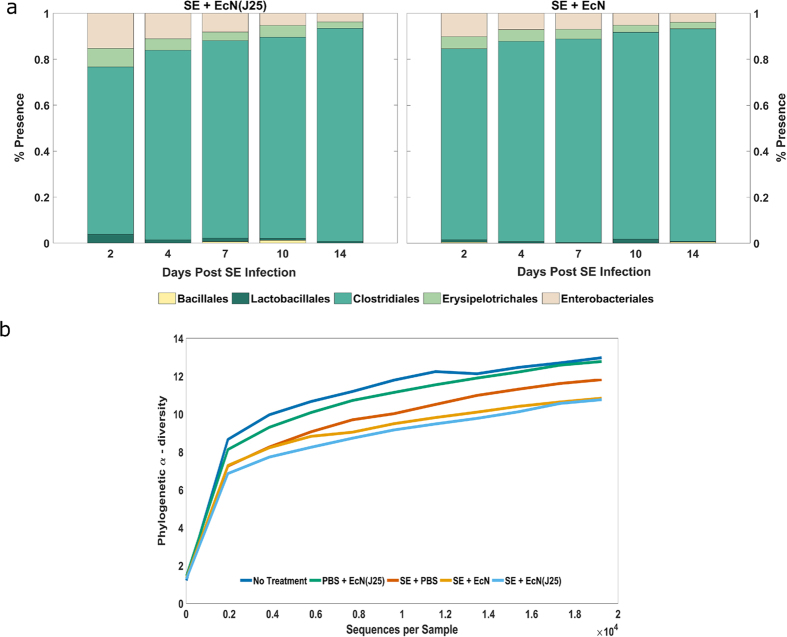
Modified probiotic has no discernible impact on microbial diversity (**a**) Presence of microbial species in the bird’s microflora in the ‘order’ taxonomic category. The ceca samples extracted from poults challenged with SE and treated with the modified probiotic (left) and unmodified probiotic (right) were averaged across both trials. The percent presence (×100) of the microbial species are shown for each collection point. (**b**) Alpha-diversity plot for Trial 1 at day 14 post infection. UniFrac phylogenetic diversity was used as a criterion.

In Fig. 4a, it is evident that microflora profiles in groups treated with the modified or unmodified EcN have no major differences. This implies that the microcin does not significantly alter the native microbial distribution, a challenge routinely encountered with standard antibiotic regimens^26^. Consistent with previous work, we observe clear temporal shifts in bacterial populations, with Clostridia spp. dominating the microflora^27^.

In accord with recent literature^28^, SE significantly reduces microbial diversity across all infected groups (Fig. 4a). The untreated control birds and those administered solely EcN(J25) have markedly similar α-diversity profiles at day 14 (Fig. 4b). The presence of the pathogen appears to have a more appreciable effect on diversity than the modified probiotic alone. In the SE-treatment models, EcN and EcN(J25) have similar effects further substantiating our conclusion. More detailed microflora analysis is available in Supplemental Fig. 8.

## Discussion

The presented technology reduced cecal SE carriage in one of the two trials, taking advantage of the remarkable character of MccJ25, a naturally occurring AMP. AMPs are ancient host defense effector peptides, produced by organisms across all biological domains as part of the innate immune response against microbial challenge^29^. AMPs, for all their promise as alternatives to antibiotics, have failed in translational success, largely due to their high synthesis costs and rapid degradation rates in the body^29^.

The use of a probiotic delivery vector may overcome these economic and transport hurdles, enabling localized production of AMPs at the site of infection. EcN has demonstrated numerous health benefits in poultry^24^,^25^ and is equipped with several fitness factors that allow it to persist in the intestinal environment, making it a prime candidate for this delivery platform^30^.

The brief period of colonization by EcN allows for a tunable treatment regime, enabling continuous administration through feed. The delivery of antimicrobial molecules may then be sustained for hours, or even days, localized at the site of infection.

Several key questions concerning the delivery of peptides by bacteria remain. The most pressing ones are perhaps the ones related to the use of modified organisms. In particular, the levels of release to the environment and the rate of DNA transfer of engineered components to other microflora species have not been studied. There are ways to mitigate these risks, including suicide genes that will destroy the calls outside host GI tracts. But the results in this study suggest that larger programs that focus on the safety of antibiotic probiotics are warranted.

From a practical viewpoint, a critical question is the influence of numerous alternative dosing regimens on SE carriage, bird health, and host microflora. A single, 1 ml inoculum was sufficient to alter the trajectory of SE carriage and expedite shedding in Trial 1. Continuous administration by incorporation in the water or feed may have the potential of rapidly clearing SE in pre-harvest poultry.

There are multiple engineering choices impacting the performance characteristics of antibiotic probiotics, including the choice of the bacterial strain and its colonization profile, the antimicrobial peptides, the expression strength, and the secretion paths, to name a few. Optimizing the efficacy of antibiotic probiotics and determining the best dosing regimen may be best addressed through a combination of carefully designed field studies and smaller scale animal experiments.

Finally, we note that the described strategy does not follow the traditional drug discovery and delivery process. Instead of identifying new therapeutic targets in pathogenic bacteria and developing new classes of drug molecules, probiotic bacteria can be recruited in the fight against pathogens. With available libraries of organisms, of AMPs, and of vectors for peptide production and secretion, this technology may be developed for numerous GI tract pathogens in a variety of hosts. Fine-tuning antibiotic probiotic systems may offer an alternative to antibiotics used in agriculture for pathogen reduction. This strategy can also potentially offer sorely needed solutions in the growing fight against antibiotic-resistant strains that infect and sicken humans.

## Materials and Methods

### Bacterial strains and plasmid construction

The Pon promoter and the ProTeOn activator protein were synthesized by GENEART in a pMS expression vector that contains a ColE1 origin of replication and a spectinomycin selection marker. Using standard molecular biology techniques, the mcjABCD operon was PCR amplified from the PJP3 vector (donated by J. Link, Chemical and Biological Engineering, Princeton University) and cloned into the pMS plasmid between the EcorI and SacI restriction sites. The final construct, pBF25, is illustrated in Supplemental Fig. 1. pBF25 was transformed into *E. coli* Nissle 1917 by electroporation for characterization (EcN(J25)).

### Zone of Inhibition Activity Assay

EcN(J25) was cultured in Luria-Bertani (LB) media at 37 °C with agitation at 225 rpm. 1 ml of duplicate overnight cultures were transferred to a 2 ml microcentrifuge tube and centrifuged for 3 min @ 2.5rcf and the pellet was washed with 0.5 ml of 1x PBS. At t = 0 hrs, the washed EcN(J25) was inoculated in 2 ml of fresh LB to an OD of 0.05 in 5 sterile culture tubes, allowing one tube per time point. At each point (0, 2, 5, 10 and 20 hrs) post-inoculation, 1.5mls of culture supernatant was collected from the respective tube and centrifuged for 1 min at 15.8rcf. The supernatant was sterile filtered and stored at 4 °C. OD600 was measured at each time point.

M9 minimal agar plates (15 g/L) were overlaid with 10 ml of soft M9 agar (6.5 g/L) containing 10E6 CFU of SE. The supernatant was two-fold serially diluted and 10 μl of each dilution was spotted on the SE-agar plates. The reciprocal of the last dilution with a visible halo was taken as the activity units of MccJ25.

### *In vitro* supernatant activity assay

Cultures of EcN(J25) and unmodified EcN were grown in LB and the supernatant was collected at 16 hrs. Exponential phase SE was diluted to 10E2 CFU/ml and transferred to a 96-well plate. Microcin-rich supernatant from EcN(J25) was applied to the SE at varying volume concentrations (0, 0.5 and 1%). The wells were blanked with spent media from the unmodified Nissle supernatant to obtain a final volume of 340 μl/well. The growth assays were obtained by shaking the plate at 37 °C and taking OD600 measurements every 15 mins in a BioTek Synergy H1 microplate reader.

### Bacterial challenge/treatment of turkey poults

All experiments were performed in accordance with relevant guidelines and regulations. This project was approved by the Institutional Biosafety Committee and by the Institutional Animal Care and Use Committee (IACUC) at the University of Minnesota. Day-of-hatch Hybrid Converter Breed Tom poults, free of vaccinations, were transported from a commercial hatchery. Birds were randomly transferred to isolator units with incandescent lighting, crinoline flooring, and *ad libitium* access to antibiotic and probiotic-free food and water. Three birds were taken to the University of Minnesota Veterinary Diagnostic lab prior to each trial to confirm they were *Salmonella*-free. The remaining birds were left in the units for a 3-day acclimation period following transport.

### Bacterial Challenge Strains

The SE and EcN challenge strains were both made resistant to antibiotics prior to inoculation so they could be recovered from the intestinal tract of the birds for enumeration. SE and EcN were both made resistant to 100 μg/ml rifampicin for this antibiotic concentration proved effective in limiting bacterial background from the poults. To differentiate the challenge strains, SE was additionally made resistant to 30 μg/ml nalidixic acid and EcN to 100 μg/ml streptomycin. On days 1 and 2 post-hatch of both trials, fecal samples were collected from each isolator unit and resuspended in PBS to make a final 10x dilution. The fecal samples were plated on the respective antibiotic agar plates to ensure there was no background bacterial growth in any of the units. Bacterial resistance was achieved using a ramping up and repeated exposure protocol^31^.

### Animal trial 1

On day 0 (4-days post hatch), 140 birds were randomly selected to make 5 groups of 28 birds, with one group for each treatment ((5 birds per treatment per time point x 5 time points) + 3 extra birds per treatment to account for potential early losses). The birds were randomized at this stage to mitigate any early microbiome diversification that may have occurred during the acclimation period. Each group of 28 birds was placed into one of five isolator units and each unit underwent a different experimental treatment. The 5 treatment groups evaluated in this study were: (1) SE-control, (2) SE + EcN(J25), (3) SE + EcN, (4) EcN(J25)-control, and (5) PBS-control.

On day 0, groups 1, 2, and 3 were orally inoculated with 10E7 CFU SE. Groups 4 and 5 were inoculated with 1 ml of 1x PBS. The birds were monitored for any signs of distress for 2 hours post-challenge. On the following day the birds were inoculated in the same fashion with 1 ml of their respective treatments, groups 1 and 5 with PBS, groups 2 and 4 with 10E7 CFU EcN(J25), and group 3 with 10E7 CFU EcN. All bacterial treatments were suspended in 1 ml PBS.

On days 2, 4, 7, 10, and 14, five birds were euthanized, weighed, and necropsied from each isolator unit. We note that we added 3 surplus birds per unit to account for natural bird losses that are common in the first few days following hatch. At the final time point any remaining birds were additionally necropsied and data was collected.

### Animal trial 2

Similar to trial 1, on day 0, birds were randomized from the isolator units they stayed in during their adjustment period to make 4 new groups of 38 birds. In this study, we used two isolator units per treatment with 19 birds per unit. The 4 treatment groups in this study were: (1) SE control, (2) SE + EcN(J25), (3) SE + EcN, and (4) SE + 0.15 mg ENR/(kg of bird weight).

On day 0, all 4 groups were orally inoculated with of 10E7 CFU SE. On day 1, groups 1–4 were treated with 1 ml PBS, 10E7 CFU EcN(J25), 10E7 CFU EcN, and 1.5 mg of ENR, respectively. On days 2, 4, 7, 10, and 14, 3–4 birds per unit (6–8 birds per treatment) were euthanized, weighed, and necropsied from each unit. At the final time point, any remaining birds were also necropsied and data was collected for all birds.

### Enumeration of *Salmonella* and Nissle in cecal contents

On days 2, 4, 7, 10 and 14 post-inoculation, birds were randomly selected and euthanized from each isolator unit. Their body weight was recorded and both ceca pouches were extracted. The ceca samples were weighed and transferred to sterile 7 ml Precellys tubes (Bertin Corp) containing ten 2.8 mm- zirconium oxide (Bertin Corp) beads. Each sample tube was processed by adding 2 ml PBS and using a Minilys^®^ homogenizer for 15 s at low speed.

The homogenate for each sample was serially diluted in 10× increments and plated on selective media for SE and EcN enumeration. The plates were incubated overnight at 37 °C and then the colonies were counted.

### Statistical analysis of cecal counts

CFU/g of ceca was determined for both SE and EcN from each bird at each time point. The data was log10 transformed and all data points with an enumerated CFU below the limit of detection were given a value of 0.5 CFU/g ceca. Extended discussion of the normality testing is available in Supplemental Fig. 9.

Trials 1 and 2 were analyzed via ANOVA separately (Table 1). We first included all experimental groups, including the enrofloxacin treatment used in Trial 2. In both trials, the ‘Treatment’ and ‘Day’ were described as categorical variables, as well as, their interactions to account for different time-dependent responses.

Given these results, the null hypothesis that all treatments had the same effect was rejected. Therefore, pairwise post-hoc analysis was done between all treatments across all times using the Tukey-Kramer method with the computed confidence intervals (CI) of the differences of the transformed data (Tables 2 and 3).

### Microbiome Extraction and Analysis

DNA was extracted from ceca samples using the MoBio Powersoil kit (Mo Bio Labs). Sequencing of the samples was performed at the University of Minnesota Genomics Center using Illumina MiSeq paired-end 2 × 300 bp technology of the V4 region of the 16S rRNA. The pair reads were assembled using PandaSeq^32^. The quality threshold for PandaSeq was set at 0.9. After the assembly, sequences were trimmed and converted from fastq to fasta format. ChimeraSlayer’s USEARCH 6.1 method^33^,^34^ was used to remove potential chimeras. For the operational taxonomic unit (OTU) picking, QIIME’s^34^,^35^ open reference method was employed. For the closed-reference OTU picking, the Greengenes library^36^ was employed through QIIME. For the unclassified sequences, de novo Uclust OTU picking^33^ was used. QIIME was also used for the alpha diversity analysis using UniFrac^37^.

### Vertebrate Animal Experiments

The University of Minnesota Institutional Animal Care and Use Committee has reviewed and approved protocol 1409-31793 A involving all live vertebrate animals described herein, ensuring compliance with federal regulations, inspecting animal facilities and laboratories and overseeing training and educational programs.

## Additional Information

**How to cite this article**: Forkus, B. *et al*. Antimicrobial Probiotics Reduce *Salmonella enterica* in Turkey Gastrointestinal Tracts. *Sci. Rep.* **7**, 40695; doi: 10.1038/srep40695 (2017).

**Publisher's note:** Springer Nature remains neutral with regard to jurisdictional claims in published maps and institutional affiliations.

## Supplementary Material

Supplementary Information

## Figures and Tables

**Table 1:** ANOVA results for Trial 1 and Trial 2.

Trial	Treatment F-value	p-value
1	3.96	0.02
2	8.19	<0.01

**Table 2:** Pairwise analysis for Trial 1 treatments.

Trial 1					
Treatment		Transformed 95% CI			p-value
i	j	Lower	Center	Upper	
PBS	EcN(J25)	0.07	0.69	1.30	0.03
PBS	EcN	−0.47	0.15	0.78	0.83
EcN(J25)	EcN	−1.15	−0.53	0.09	0.11

**Table 3:** Pairwise analysis for Trial 2 treatments.

Trial 2					
Treatment		Transformed 95% CI			p-value
i	j	Lower	Center	Upper	
PBS	EcN(J25)	−0.12	0.29	0.69	0.27
PBS	EcN	−0.24	0.17	0.57	0.71
PBS	ENR	−0.88	−0.46	−0.05	0.02
EcN(J25)	EcN	−0.52	−0.12	0.29	0.87
EcN(J25)	ENR	−1.17	−0.75	−0.33	<0.01
EcN	ENR	−1.05	−0.63	−0.22	<0.01
