# Supplementary material for: Antimicrobial Probiotics Reduce Salmonella enterica in Turkey Gastrointestinal Tracts
Source: Sci Rep. 2017 Jan 17;7:40695. doi: 10.1038/srep40695 (PMC5240571; doi:10.1038/srep40695)
Supplement: Supplementary Information [file srep40695-s1.pdf]

Supplemental Information

Antimicrobial Probiotics Reduce *Salmonella* enterica in Turkey Gastrointestinal Tracts

Brittany Forkus[1], Seth Ritter[1], Michail Vlysidis[1], Kathryn Geldart[1] and Yiannis N. Kaznessis[1,*]

[1]Department of Chemical Engineering and Materials Science

University of Minnesota, Minneapolis, MN 55455, USA

[*]Correspondence and requests for materials should be addressed to Y.N.K (email: yiannis@umn.edu)

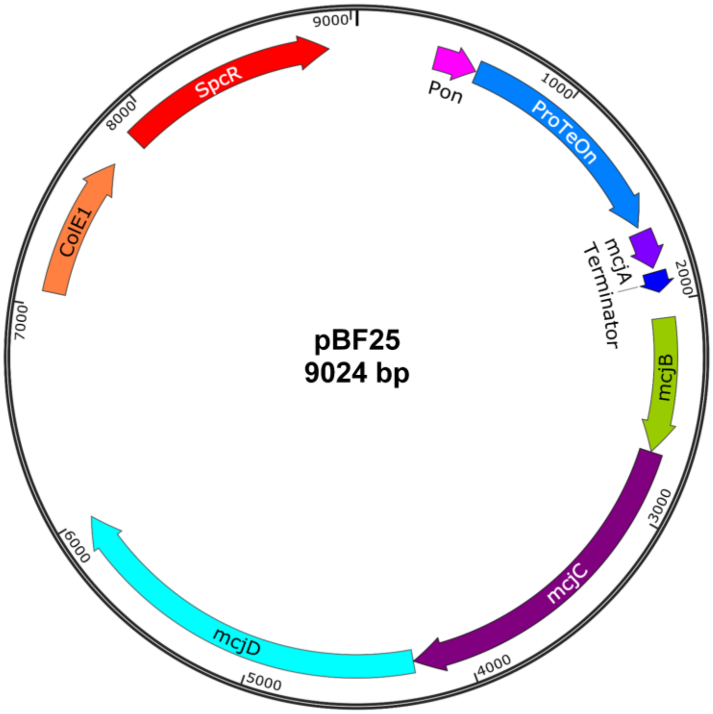

Supplemental Figure 1**. Plasmid map of pBF25**. pMS expression vector containing a ColE1 origin of replication and spectinomycin selection marker. mcjABCD was cloned from PJP3 between EcorI and SacI restriction sites via standard molecular cloning procedures to create pBF25. Figure made with SnapGene Viewer.

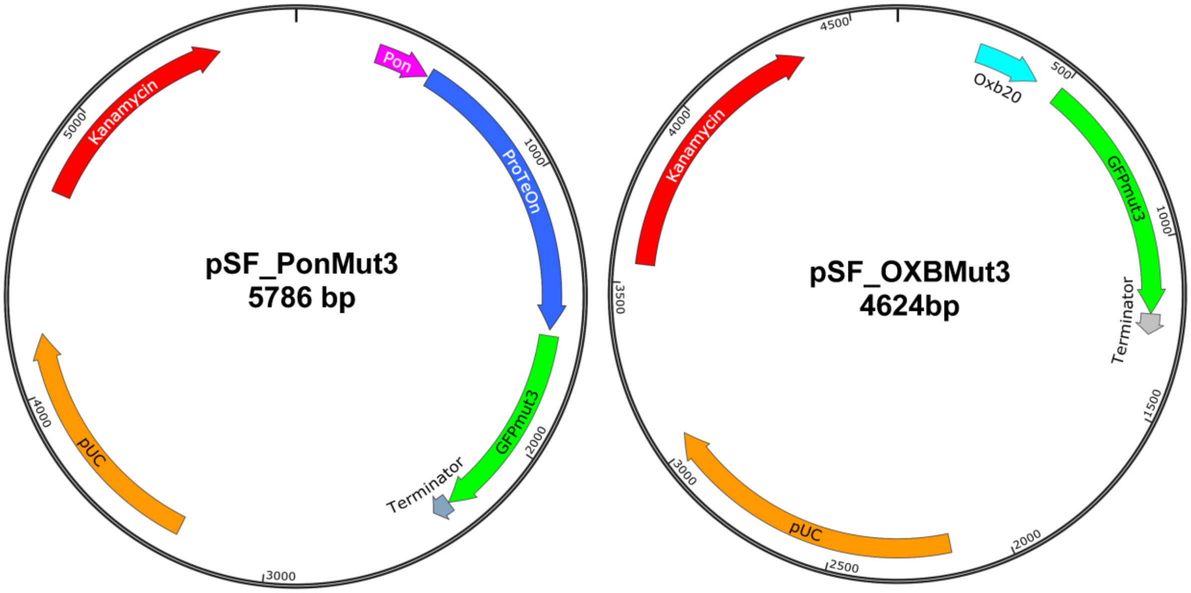

Supplemental Figure 2. **Plasmid maps for pSF_Oxbmut3 and pSF_PonMut3**. Both plasmids have a pSF backbone (Oxford Genetics) containing a pUC origin or replication and a kanamycin selection marker. The Oxb20 and ProTeOn+ promoters were used to drive expression of GFPmut3 in each respective vector. Both plasmids were created using standard molecular cloning techniques. Figures were made using SnapGene Viewer.

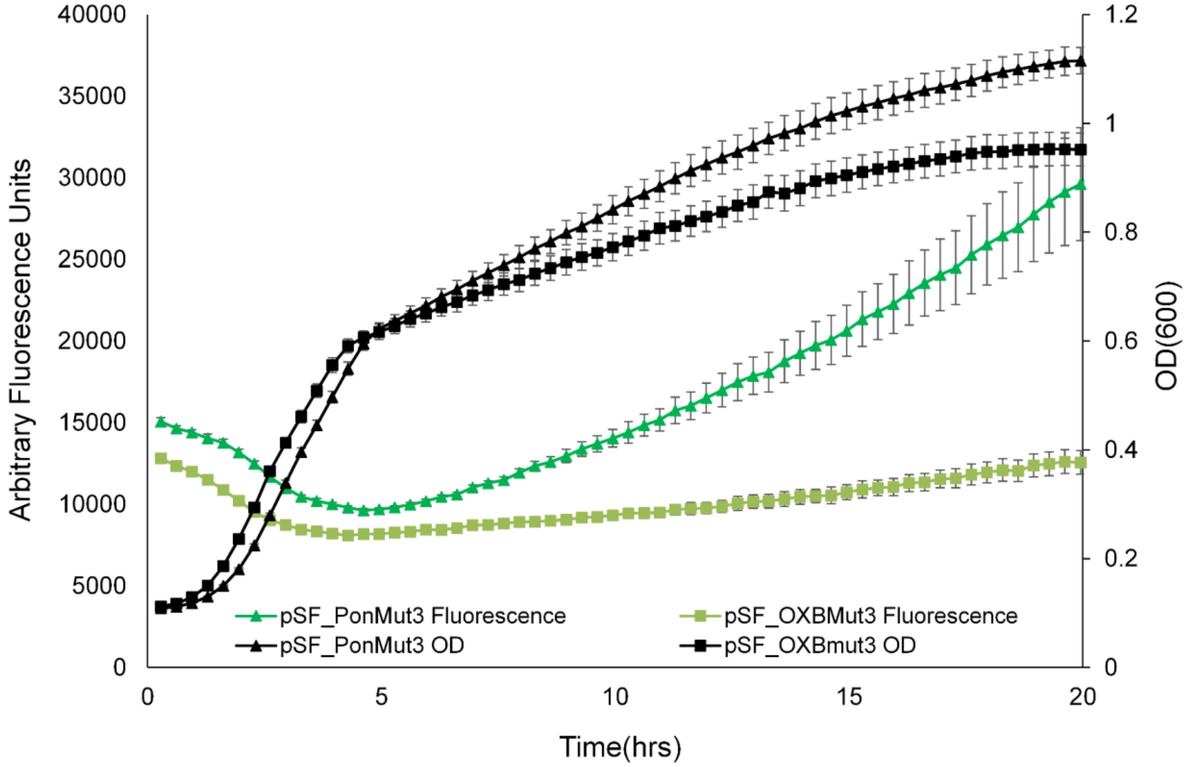

Supplemental Figure 3. **GFP assay for comparison of promoter strength**. pSF_OXBMut3 and pSF_PonMut3 plasmids were both transformed into chemically competent T7 express cells (New England Biolabs). Both strains were cultured overnight in LB media supplemented with 50µg/ml kanamycin. The stationary phase cultures were diluted to an OD 0.5 in fresh LB media and 10µl was transferred to a clear bottom, black 96-well plate (CellVis) in 4 replicates. Selective LB was added to each well to a final volume of 340µl/well. The plate was shaken in double orbital mode in a BioTek Synergy H1 microplate reader at 37°C for 20 hrs taking OD(600) and fluorescence measurements (Ex 485, Em 528, gain 100) on 15min intervals.

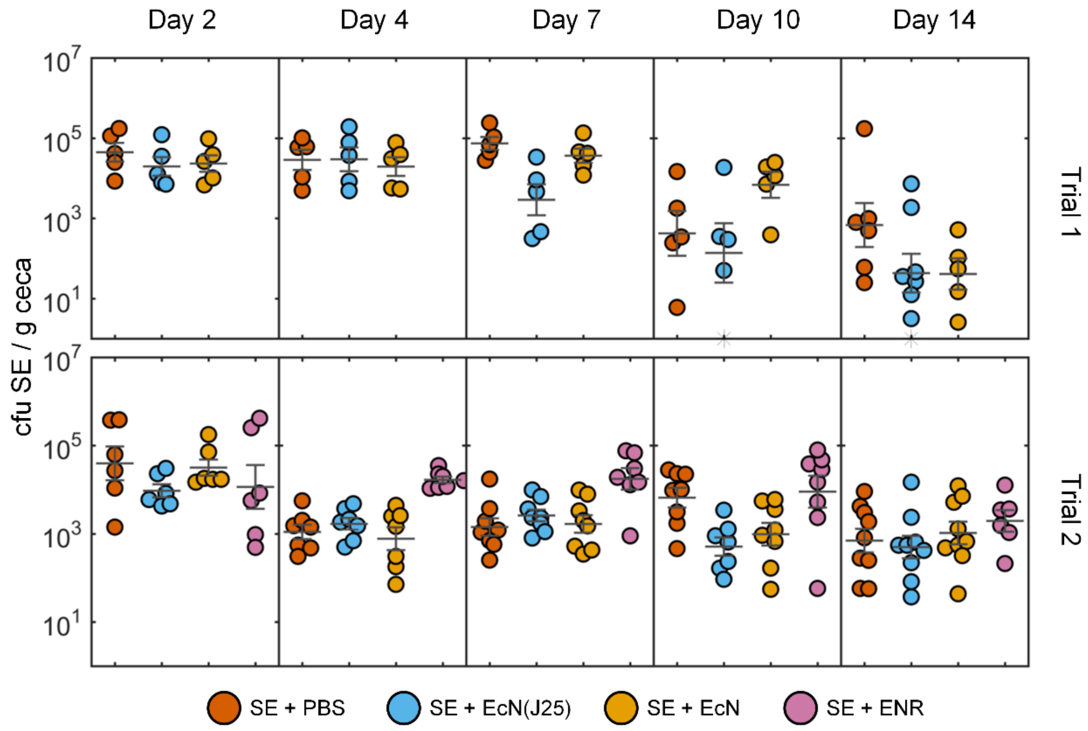

Supplemental Figure 4. **Trajectories of individual bird SE counts**. The charts show the SE counts in the ceca for each individual bird at each collection point. The top row shows data collected for Trial 1 and the bottom shows the same data for Trial 2.

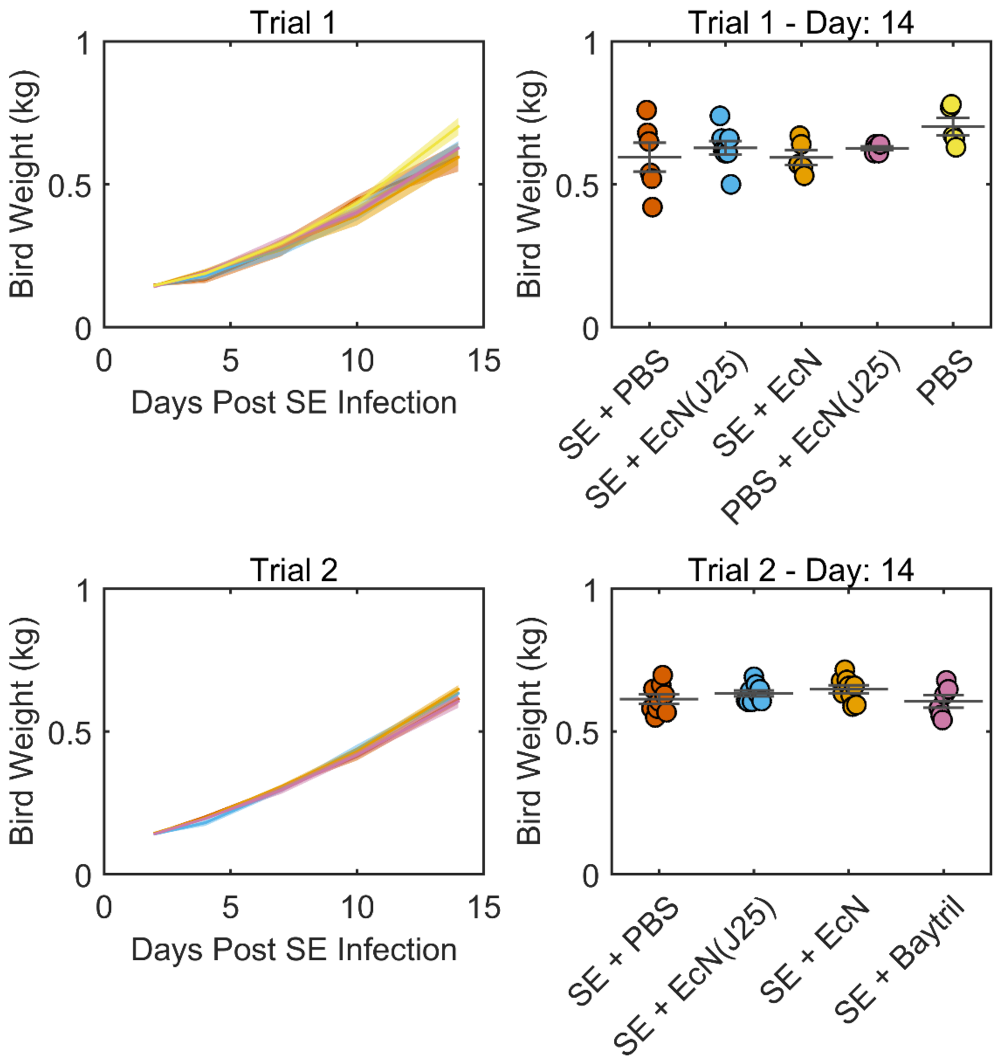

Supplemental Figure 5. **Bird weight profiles of *in vivo* trials**. The left column shows the average bird weights of the euthanized birds at each time point for each respective trial. The right column shows the bird weights for the individual birds euthanized at the final time point, 14 days post SE-challenge. The width of the lines and error bars correspond to the standard error and the color represents the treatment group. The groups show no statistical difference in average bird weight across all time points.

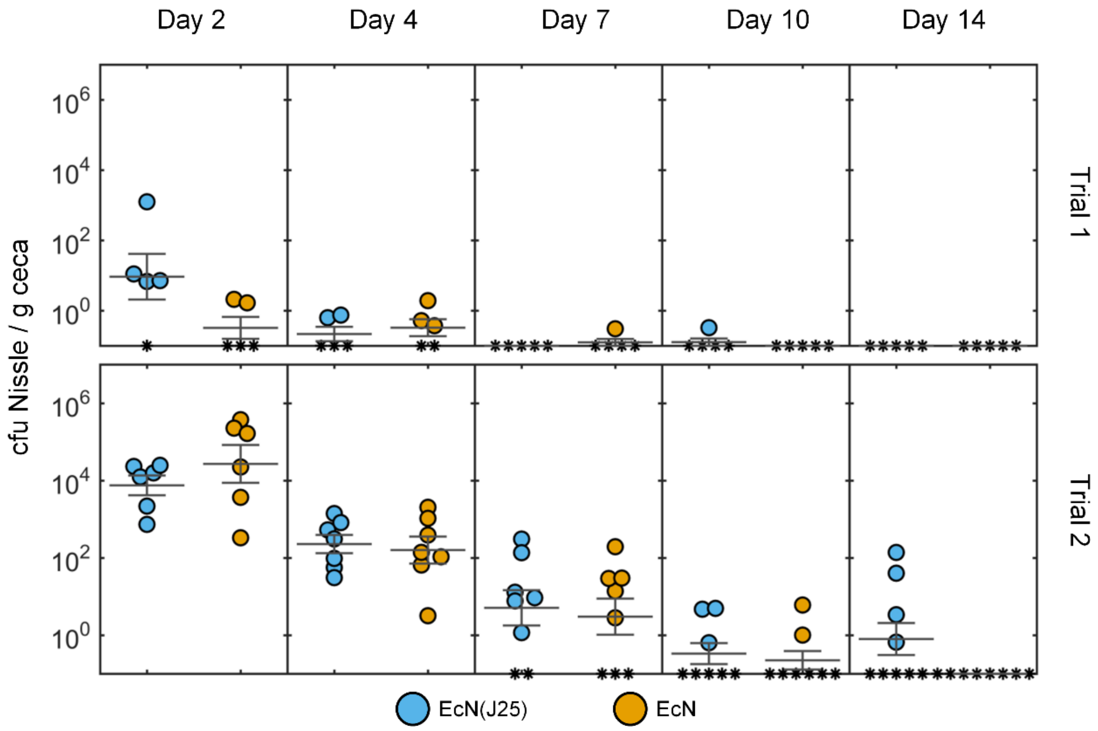

Supplemental Figure 6. **Trajectories of individual bird Nissle counts**. The charts show the Nissle counts in the ceca for each individual bird at each collection point. The top row shows data collected for Trial 1 and the bottom shows the same data for Trial 2. Error bars represent standard error. Samples below limit of detection are plotted on the x-axis.

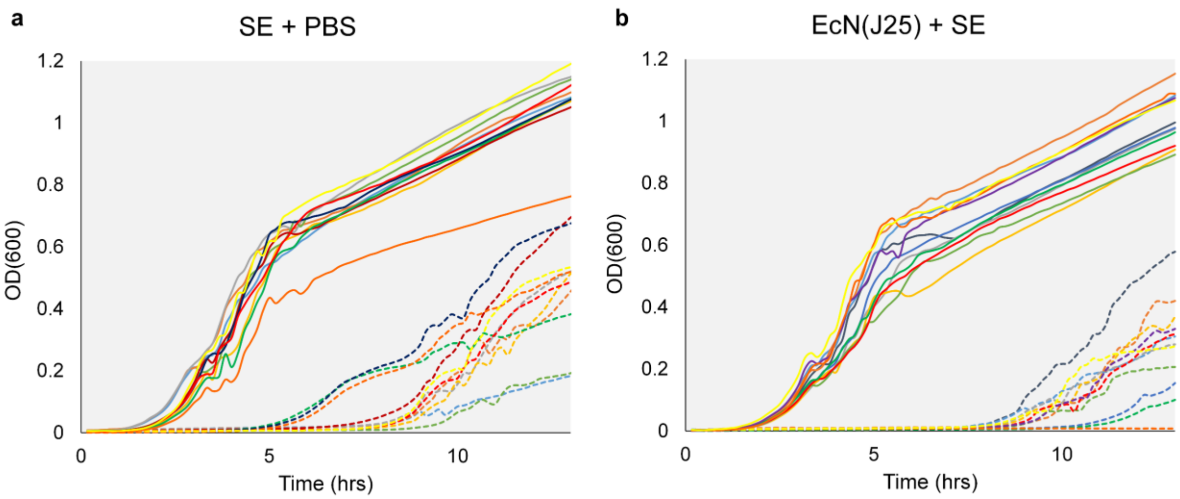

Supplemental Figure 7. **SE-susceptibility to MccJ25 following GI passage**. 12 SE isolates were collected from birds treated with (a) SE+ PBS and (b) EcN(J25) + SE after passage to the ceca on day 14 post challenge to determine if any resistance was developed to MccJ25. The isolates were challenged with 0 and 20% by volume of EcN(J25) supernatant that was collected after 20hrs of growth. Kinetic growth curves were generated using the supernatant activity assay described in the materials and methods. Same color refers to the same isolate, solid line is growth in absence of MccJ25 and dotted line is growth in the presence of MccJ25. No resistance is observed in any of the 12 isolates in either group as clear growth inhibition is observed across all isolates in both treatment groups.

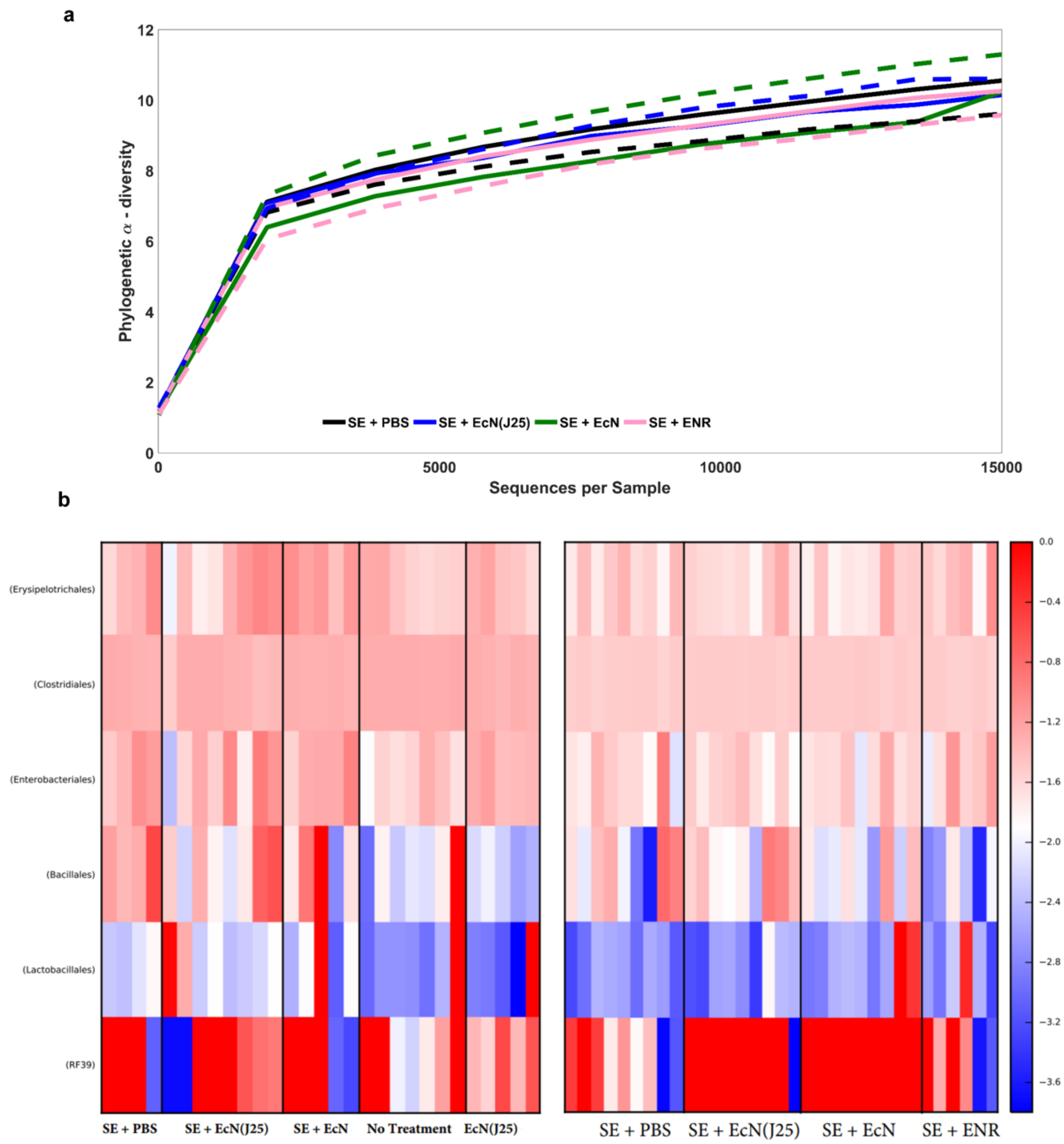

Supplemental Figure 8. **Microbiome diversity at final time point** (a) Alpha diversity of all treatment groups in Trial 2. (b) Heat map comparing the frequencies of major bacterial species for each bird necropsied at the final time point for all treatment groups in (left) Trial 1 and (right) Trial 2. Each column represents an individual bird.

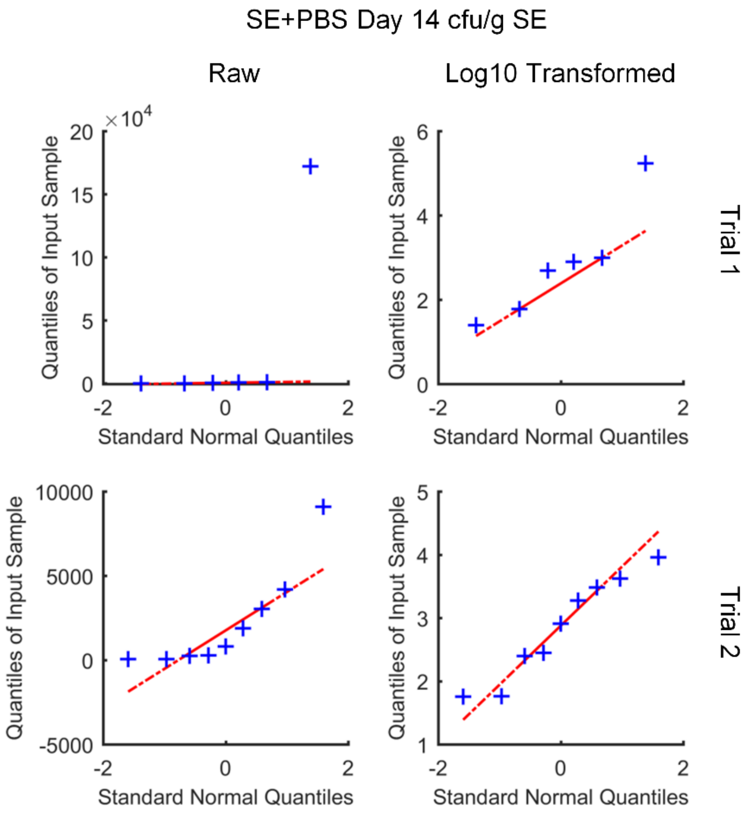

Supplemental Figure 9. **Quantile-Quantile plot for comparing raw and log10 transformed data for normality.** A Q-Q plot enables the determination of whether a distribution assumption is valid. The SE counts from day 14 in both Trial 1 (top) and 2 (bottom) were tested for normality using the raw (left) and transformed (right) values. The transformed data appears to yield a better approximation.
